# Emerging opportunities in ROP therapy: from inhibiting pathological vessel growth to promoting physiological vascularization

**DOI:** 10.3389/fmed.2025.1716449

**Published:** 2026-01-12

**Authors:** Luca Filippi, Alessandro Pini, Paola Bagnoli, Massimo Dal Monte, Maurizio Cammalleri

**Affiliations:** 1 Department of Clinical and Experimental Medicine, University of Pisa, Pisa, Italy; 2 Department of Experimental and Clinical Medicine, University of Florence, Florence, Italy; 3 Department of Biology, University of Pisa, Pisa, Italy

**Keywords:** angiogenesis, beta blockers, oxygen, proliferative retinopathy, vascularization

## Abstract

Despite continuous advances in the care of preterm infants, therapeutic management of ROP has made limited progress in recent years and remains a source of frustration for neonatologists. The current approach largely relies on the spontaneous resolution of the disease, limiting clinical intervention to observation and monitoring, with little capacity to significantly intervene on the pathological progression of retinal vascularization. Similarly, ophthalmologists often adopt a watchful waiting strategy, with invasive treatments reserved only for preventing imminent retinal detachment. As a result, ROP remains an orphan disease in terms of targeted pharmacological therapies that address its underlying pathophysiological mechanisms.

However, recent years have brought significant advances in understanding the biological mechanisms that regulate retinal vascularization, pointing to the catecholaminergic stimulation of specific β-adrenoceptors (β-ARs). Indeed, after birth, β2-ARs appear to play a predominant role in coupling hypoxia to excessive vascular growth in the proliferative phase of ROP as it occurs in infantile hemangioma in which β2-AR blockade with propranolol, a non-selective β-AR antagonist, is the treatment of choice to prevent chaotic vessel proliferation. In this line, propranolol-based ophthalmic solutions may offer a promising balance of efficacy and safety in ROP. However, preclinical studies have shown that β2-AR blockade with propranolol suppresses pathological vascularization without promoting vessel regrowth in the avascular area. Additional therapeutic opportunities can be provided by our project regarding the role of β3-AR activation in promoting the revascularization of the central retina otherwise vaso-obliterated in response to hyperoxia, through recovered astrocyte template, which is likely to play an important role in vasculature recovery.

This possibility paves the way for preventive pharmacological strategies using β3-AR agonists against ROP. It is likely that in the coming years an approach similar to that leading to explore the potential of propranolol in ROP, might be used to extend to preterm infants the results of preclinical studies on the efficacy of β3-AR agonism. In this case, the goal would be to stimulate the physiological process of vascularization rather than to slowing down the progression of ROP.

## Introduction

In recent decades, significant progress has been made in the care of preterm infants, leading to a substantial reduction in neonatal mortality, even among those with extremely low gestational age, and to marked improvements in major neonatal outcomes (1). One of the fields where the most remarkable advances have been achieved concerns the respiratory outcomes of preterm infants: a series of clinical strategies (reduced use of invasive mechanical ventilation and the adoption of gentler ventilation techniques) and pharmacological interventions (antenatal steroid prophylaxis, appropriate use of surfactant) have radically transformed the clinical course of bronchopulmonary dysplasia (BPD), the main chronic pulmonary disease of prematurity. These advances have been so profound that they necessitated a redefinition of the disease, leading to the current designation of “new BPD” as opposed to the historical “old BPD” (2).

Such improvements have been obtained through a deeper understanding of the pathophysiological mechanisms responsible for pulmonary injury, with neonatologists directing their interventions specifically toward counteracting these mechanisms.

In contrast to these achievements in neonatal respiratory care, the role of the neonatologist in preventing retinopathy of prematurity (ROP) has not shown comparable progress in recent years. At present, the preventive strategies available to neonatologists remain largely limited to more cautious oxygen administration, since excessive oxygen exposure during the early postnatal weeks is recognized as the *primum movens* of arrested retinal vascularization (the first phase of ROP), subsequently leading to hypoxia and the induction of pathological neovascularization (the second phase of ROP). Interventions that may limit the development of ROP include the promotion of human milk feeding, maternal supplementation with omega-3 fatty acids during lactation, and the administration of vitamin A to preterm infants (3). In practice, neonatologists often rely on the spontaneous resolution of the disease, limiting themselves to monitoring its course without being able to significantly influence the progression of pathological retinal vascularization. Consequently, the current neonatal strategy for ROP can be reasonably described as frustrating.

Similarly, ophthalmologists adopt a wait-and-see approach. They are aware that, during the early postnatal weeks, exposure to a relatively hyperoxic environment impedes retinal vascularization. For this reason, they avoid performing ophthalmic examinations at this stage, as these would only reveal an avascular retina classified as “immature,” while also causing discomfort to the infant (4). However, ophthalmologists currently lack therapeutic tools capable of reactivating the physiological process of retinal vascularization that would have naturally occurred if the fetus had remained in the hypoxic intrauterine environment. Thus, the first ophthalmic examinations are generally performed at around 32−33 weeks’ postmenstrual age, after which the infant undergoes serial ophthalmological evaluations aimed at detecting the onset of pathological vascularization (5). This latter, unlike physiological vascularization - which is characterized by linear, centrifugally oriented vessels with an effective blood-retinal barrier - is instead marked by irregular, tortuous vessels extending toward the vitreous body, accompanied by an immature and functionally ineffective barrier (6).

Throughout this period, neither neonatologists nor ophthalmologists have access to therapeutic strategies capable of slowing the progression of pathological vascularization. Similarly, no interventions that can foster the development of a physiological vascular network are still available. The only available ophthalmic interventions are invasive, aimed at preventing imminent retinal detachment, through either laser photoablation of ischemic retina or intravitreal administration of anti-Vascular Endothelial Growth Factor (VEGF) agents (7).

Therefore, ROP currently remains an orphan disease with respect to specific pharmacological therapies targeting its underlying pathophysiological mechanisms. Available interventions are limited to emergency treatments designed to prevent retinal detachment.

## Pathogenesis

In recent years, substantial progress has been made in elucidating the biological mechanisms that regulate both physiological and pathological retinal vascularization. Central to this process is the role of oxygen. The low oxygen level characteristic of intrauterine life is responsible for maintaining elevated concentrations of hypoxia-inducible factor 1 (HIF-1) (8), a key transcription factor that activates the expression of approximately 200 genes, several of which are directly involved in physiological angiogenic processes (9). Among these, VEGF - a primary HIF-1 target gene - plays a pivotal role in the induction of physiological retinal vascularization, which in humans begins around the 16th week of gestation (10).

It is widely accepted that hypoxia, elevated HIF-1 levels, and increased VEGF expression are essential for the promotion of retinal vascularization (11). At the same time, it is well established that the development of retinal vascularization follows an astrocytic template, which is induced by hypoxia and HIF-1 (12), while also representing an important source of VEGF (13). Accordingly, the currently available evidence suggests that intrauterine hypoxia primarily induces the maturation of the astrocytic network, which in turn contributes to VEGF production and thereby to the initiation of vascularization. However, these factors alone do not appear to be sufficient to trigger physiological vascularization. In fact, data available from rodents indicate that during intrauterine life, when the fetus is in a state of physiological hypoxia and both HIF-1 and VEGF levels are markedly elevated (14), retinal vessels remain rudimentary or absent (15). This indicates that HIF-1 and VEGF alone are necessary but non-sufficient to initiate vascularization. On the contrary, in rodents, retinal vessels appear after birth (15), when exposure to higher oxygen concentrations leads to a reduction in HIF-1 and VEGF (14). Therefore, experimental evidence from animal models suggests that physiological vascular development is modulated by oxygen gradients and, at least in rodents, follows a biphasic trend (16): an initial intrauterine phase, characterized by marked hypoxia, is required to promote the astrocytic scaffold and the recruitment of endothelial progenitor cells (EPCs) (17); however, the development of retinal vessels requires a subsequent increase in oxygen levels, with a concomitant reduction in HIF-1 and VEGF. This finding aligns with evidence that human endothelial progenitor cells preserve stemness exclusively under hypoxic conditions, whereas normoxic exposure promotes their differentiation (18), a phenomenon also described in human embryonic stem cells (19).

This mechanism suggests that, while *in utero*, the retina of rodents already possesses an astrocytic scaffold and has largely recruited EPCs, which differentiate only after birth as hypoxia decreases. Such a mechanism would enable EPCs to act selectively where and when needed - namely in hypoxic and damaged tissues - differentiating once exposed to higher oxygen levels; in this way, new vessels would preferentially form adjacent to pre-existing ones. This oxygen-dependent maturation process could explain the centrifugal progression of retinal neovascularization, as only EPCs located near pre-existing vessels – and thus exposed to higher oxygen tension - would differentiate into mature endothelial cells.

Beginning around the 16th week of gestation, retinal human vascularization continues until term (10). Observations from rodent models, where a biphasic oxygen pattern governs retinal vascularization, prompted investigations into whether a similar biphasic pattern could be observed in human fetal oxygenation. Data from over 4,000 cord blood gas analyses performed at birth in neonates of varying gestational ages (representing intrauterine oxygenation status) confirmed this biphasic trend (20–22). From approximately 22 weeks of gestation onward (earlier data are unavailable), the human fetus becomes progressively more hypoxic, reaching maximal hypoxia at 33–34 weeks (21, 22). This progressive decline in oxygenation is likely attributable to placental growth, which increases oxygen consumption and thus reduces oxygen availability for the fetus. During this period, it is reasonable to infer that HIF-1 and VEGF levels rise progressively, promoting astrocytic scaffold formation and EPC recruitment. A second phase follows, characterized by increasing oxygen levels (20, 22). Unlike rodents, however, in humans this second phase also takes place *in utero*, beginning at around 33–34 weeks of gestation. It is presumably associated with placental senescence, which reduces the efficiency of the placental barrier, allowing greater transplacental passage of oxygen as well as other components critical to fetal well-being, such as immunoglobulins.

In summary, oxygen plays a determinant role in physiological retinal vascularization: in the initial phase, reduced oxygen tension increases HIF-1 and VEGF expression, promoting astrocytic scaffold recruitment and EPC mobilization; subsequently, increased oxygen availability supports their orderly and centrifugal maturation.

Whether oxygen exerts these effects exclusively through modulation of HIF-1 and VEGF, or also through additional intermediaries, has been the principal focus of research in recent decades.

## Role of β-adrenergic receptors

It has long been established that hypoxia induces a significant release of catecholamines, representing a protective response against oxygen deprivation (23). One of the main mechanisms to counteract hypoxia is the induction of vascularization, and catecholamine release represents a key trigger of this process, both in physiological contexts such as revascularization of ischemic regions (24, 25), and in pathological settings such as tumor vascularization (26). Studies have focused on the role of β-adrenergic receptors (β-ARs), identifying β2-AR as the receptor most critically involved (27).

Over the past decade, compelling evidence has emerged that in the retina, hypoxia, through the upregulation of HIF-1, promotes pathological angiogenesis not only by inducing VEGF but also via the release of catecholamines and their interaction with specific β2-ARs (28, 29).

The involvement of the adrenergic system in the pathogenesis of ROP was initially investigated in the mouse model of oxygen-induced retinopathy (OIR), which represents a valuable system for elucidating the complex relationship between oxygen levels and retinal vascular modifications, as well as for reproducing the biphasic progression of ROP in humans (30). On postnatal day 7, when retinal vascularization is still incomplete, newborn pups and their nursing mother are exposed for 5 days to high oxygen concentrations (approximately 75%). This hyperoxic environment leads to the downregulation of pro-angiogenic factors and induces extensive vaso-obliteration around the optic nerve head (first ischemic phase). This initial step of the model mimics the condition observed in premature infants during the first weeks of life, which ophthalmologists describe as an “immature” or “avascular” retina. After these 5 days of hyperoxia, the pups and their mother are returned and maintained under normoxic conditions for an additional 5 days. This abrupt transition to lower oxygen availability is perceived by the mouse retina as relative hypoxia, triggering HIF-1-mediated neovascularization (30). The ensuing revascularization does not involve the central retina, which remains avascular, and markedly differs from physiological vascular development, giving rise to tortuous vessels that extend three-dimensionally into the vitreous body, with a severely compromised blood-retinal barrier. This process closely resembles the pathological vascular proliferation observed in infants during the proliferative phase of ROP, which develops at the boundary between the avascular peripheral zone and the inner retina that remains partially vascularized (30). In mice, this intense vascular proliferation may result in hemorrhages and vitreous edema owing to the immaturity and excessive permeability of the neovessels (31); in humans, the clinical course can be even more severe, due to the risk of fibrotic tissue formation and consequent retinal traction. Using this model, it was possible to demonstrate that the tight correlation between oxygen levels and vascular modulation is mediated through catecholamine release and interaction with the β-adrenergic system (28, 29).

The hypothesis that the β-adrenergic system - and more specifically β2-ARs - was involved in the coupling mechanisms between hypoxia and vascularization originated from the serendipitous clinical observation in humans that treatment with propranolol, a non-selective β1/β2-AR blocker, led to regression of infantile hemangiomas (IH) (32), the most common benign vascular proliferative lesion in infants, typically triggered by preexisting ischemic and hypoxic conditions (33). IH and ROP are frequently associated in preterm neonates (34, 35) and numerous analogies have been described between the development of IH and the onset of the proliferative phase of ROP (36, 37). The striking similarities between IH vascularization and the proliferative phase of ROP prompted a series of investigations, initially in animal models and subsequently in clinical settings, aimed at elucidating the role of β2-ARs in pathological retinal angiogenesis and evaluating the potential therapeutic efficacy of propranolol in ROP.

## Preclinical studies on OIR

Experimental studies using the OIR model have provided robust evidence regarding the role of β2-ARs in hypoxia-driven pathological angiogenesis and the potential efficacy of treatment with propranolol. Pharmacological blockade of β2-ARs administered during the hypoxic phase significantly reduced pathological neovascularization by preventing HIF-1 upregulation, attenuating tuft formation, and downregulating multiple proangiogenic factors (28, 29). This effect was attributable to the inhibition of the interaction between catecholamines, which are markedly upregulated in the OIR retina, and β2-ARs (38). Importantly, the antiangiogenic action of propranolol was selective for pathological neovascularization, as β2-AR inhibition did not induce significant alterations in tissues undergoing physiological angiogenesis (28).

Beyond its vascular effects, propranolol also exhibited neuroprotective properties: in OIR models, it preserved retinal function by enhancing autophagy, inhibiting apoptosis (39), and preventing astrocyte degeneration (40), thereby mitigating visual dysfunction associated with neuronal damage. Collectively, these findings indicate that propranolol exerts both antiangiogenic and neuroprotective effects, an unusual outcome since the reduction of neovascularization does not necessarily correlate with the recovery of retinal function (41).

## Clinical studies on ROP

Based on preclinical evidence, oral propranolol was tested in preterm infants with ROP during the proliferative phase of the disease through several pilot trials, showing efficacy but raising safety concerns due to systemic side effects (42–50). Four meta-analyses confirmed that oral propranolol slows retinal neovascularization and reduces the need for laser or anti-VEGF therapy (51–54). However, to overcome the issue of systemic toxicity, topical administration was explored. A first study confirmed the efficacy of propranolol in counteracting pathological neovascularization when administered topically in the OIR model (55); a subsequent preclinical study in healthy rabbits reported a favorable retina-to-plasma drug distribution compared with oral administration (56). Exploratory clinical trials in infants subsequently showed that propranolol eye drops (0.1–0.2%) were safe (57–59), with the higher dose significantly reducing progression to severe ROP (58, 59). These findings support further dose optimization, with the realistic therapeutic goal of reducing ROP progression by approximately 60%, consistent with meta-analytic data (51–54) and with the efficacy previously observed in IH (60).

Overall, these studies demonstrate that, in neonates, it is possible to pharmacologically uncouple localized hypoxia - whether cutaneous, as in IH, or retinal, as in ROP - from the induction of aberrant and non-physiological neovascularization, by counteracting the mediating role of the adrenergic system (16). This opens a new potential therapeutic opportunity for diseases in which pathological angiogenesis is triggered by hypoxia.

## Limitations of propranolol treatment

Despite the efficacy, safety, low cost, and ease of topical propranolol treatment in counteracting pathological vascularization in ROP, this therapeutic approach presents several limitations (61).

First, the number of clinical studies performed to date remains limited, and randomized controlled trials (RCTs) specifically designed to assess efficacy of this treatment are still lacking. Moreover, it is not yet clear whether the 0.2% concentration represents the optimal dosage or whether higher doses might enhance therapeutic effectiveness. A major obstacle to conducting RCTs is the absence of commercially available ophthalmic propranolol formulations; consequently, obtaining regulatory authorization for clinical trials is both difficult and costly. For this reason, it is desirable that future studies explore the efficacy of other commercially available non-selective β-blockers. In this respect, based on results from numerous studies (62–64) a project using timolol, a non-selective β1/2-AR blocker that is commonly prescribed as eye drops to treat glaucoma by reducing intraocular pressure, has been recently started to evaluate whether β1/2-AR blockade through the application of topical timolol may lead to comparable results as those obtained with propranolol eye drops in mice undergoing the OIR protocol.

The most scientifically relevant limitation - clearly demonstrated in the OIR model - is that β2-AR blockade is effective exclusively in suppressing pathological neovascularization, without exerting any influence on the regrowth of physiological vessels regressed following hyperoxic exposure. In fact, all animals subjected to the OIR model initially exhibit a central avascular zone, reflecting regressed vasculature induced by hyperoxia, followed by a tumultuous pathological vascularization in the peripheral retina, reflecting later hypoxia (30). Pharmacological β2-AR blockade can positively impact the abnormal vascularization but no restoring central physiological vascularization (28, 29, 38, 55).

The negligible involvement of β2-ARs in physiological angiogenesis is further supported by the observation that β2-AR blockade, as previously mentioned, does not produce significant effects in districts physiologically vascularized and devoid of pathological angiogenesis (28). Similarly, pharmacological β2-AR agonism has failed to induce any significant effect on physiological vascularization (38). Collectively, these findings confine the role of β2-ARs to hypoxia-induced pathological angiogenesis and to reparative revascularization processes, such as those occurring after birth.

Additional evidence arguing against β2-ARs involvement in physiological retinal vascular development is the finding that, during intrauterine life, when the foundations of physiological vascularization are established, β2-ARs are only weakly expressed in the retina (14).

## Analogies and differences between physiological and pathological vascularization

Physiological and pathological retinal vascularization share several initiating mechanisms: both are triggered by hypoxia, both involve upregulation of HIF-1, and both are secondary to increased VEGF expression (65). Nevertheless, their outcomes are profoundly different.

Physiological vascularization follows an orderly, linear, and centrifugal pattern, supported by a well-structured astrocytic scaffold (66), which ensures the establishment of a functional blood-retinal barrier (67). Detailed insights into this process reveal that, before the first vascular network is formed, the retina is invaded by astrocytic cells that assemble into a honeycomb-like meshwork, providing a scaffold for the subsequent alignment of endothelial cells. In the murine model, the first astrocyte precursors appear in the retina between embryonic day 12 and 14 (68), likely guided by pre-existing retinal ganglion cells (RGC) (69). In the following days, these precursors spread through the nerve fiber layer toward the retinal periphery, undergoing progressive differentiation along their trajectory; this process continues until birth (70). By birth, retinal astrocytes cover more than half of the retinal surface, extending well beyond the centrally vascularized area (71). After birth, blood vessels begin to form on the inner retinal surface, reaching the periphery within approximately 1 week (71).

Emerging evidence indicates that the molecular mechanisms guiding astrocyte recruitment and assembly into the retinal astrocytic template rely on a coordinated network of hypoxia-responsive signaling pathways. A central driver is the paracrine action of platelet-derived growth factor A-chain (PDGF-A), produced by RGC axons and acting on PDGFRα-expressing astrocytes (72). Retinal hypoxia, through HIF-1α stabilization, upregulates PDGF-A in RGCs and thereby promotes astrocyte proliferation and directed migration during retinal colonization (12). In parallel, the transcription factor PAX2 is essential for astrocyte specification, differentiation, and maturation, and contributes to their migratory competence (73). Although hypoxia-dependent regulation of PAX2 has been described in other cellular contexts, direct evidence for HIF-mediated induction in retinal astrocytes is still lacking, despite the presence of several putative hypoxia-responsive elements in the PAX2 promoter (74). Additional downstream regulators further refining this developmental program include SOX9, a master determinant of the glial lineage, which cooperates with PDGFRα signaling to maintain astrocyte identity and support their orderly arrangement within the emerging scaffold (75). Together, these pathways constitute an integrated cascade that orchestrates astrocyte recruitment and the establishment of a functional astrocytic template.

In contrast, pathological vascularization is disorganized, characterized by vascular tufts, a loss of spatial orientation with aberrant growth into the vitreous body, and disruption of the blood-retinal barrier (6). Studies conducted in the OIR model have shown that pathological neovascularization is closely associated with hyperoxia-induced astrocytic damage (76), whereas preservation of the astrocytic scaffold is essential for ensuring physiological revascularization (77). The pivotal role of astrocytes in supporting physiological vascular growth is further underscored by the finding that intravitreal injection of astrocytes prevents the onset of pathological vascularization in the OIR model. Thus, pathological vascularization may arise either from astrocyte loss induced by exposure to hyperoxia, which prevents the establishment of a guiding template for vascular regrowth (77), or from an excessive and disorganized accumulation of astrocytes during the subsequent hypoxic phase, which leads to the emergence of disorganized vessels and vascular tufts (78). Consistent with this hypothesis, histopathological analyses of human ROP specimens reveal aggregates of astrocytes at the boundary between vascularized and avascular retinal regions, suggesting disruption of their centrifugal migration (79).

In light of these considerations, several elements emerge that shed new insight on the analogies and differences between physiological and pathological vascularization. Although both processes are driven by hypoxia and involve activation of the HIF-1/VEGF axis, pathological vascularization is additionally promoted by catecholamine release and β2-AR activation (28, 29), while failing to adequately engage the astrocytic scaffold that underlies physiological vascularization. This phenomenon is clearly illustrated by the differential response of two mouse strains to the OIR model: the albino BALB/cByJ and the pigmented C57BL/6J. Both strains exhibit vascular regression following hyperoxic exposure; however, only the C57BL/6J strain develops pathological neovascularization during the subsequent hypoxic phase, whereas the BALB/cByJ strain recovers with a normal vascular pattern. In addition, in C57BL/6J mice, astrocytes begin to degenerate at the end of the hyperoxic phase and are almost completely absent after 48 h of normoxia. In contrast, astrocytes in BALB/cByJ mice survive the hyperoxic insult and can therefore guide vascular regrowth during the hypoxic phase in a manner that closely resembles physiological angiogenesis, devoid of vascular tufts (77). This finding highlights the critical role of astrocytic integrity in determining whether retinal vascularization proceeds along a physiological or pathological trajectory.

By contrast, much less is known about how physiological vascularization of the retina proceeds *in utero* - specifically, whether it also requires adrenergic signaling and how it successfully recruits the astrocytic network. This question has become particularly pressing following the recent discovery that, prior to birth, the predominant β-AR subtype expressed in the retina is the β3-AR, which is also capable of activating the pro-angiogenic pathway in the retina (14). These findings raise the possibility that the differential involvement of β-AR subtypes may contribute to the morphological and functional differences between physiological and pathological angiogenesis. More precisely, one may ask whether β3-ARs are primarily implicated in intrauterine physiological vascularization and therefore in the recruitment of astrocytes within the developing retina.

## Role of β3-adrenergic receptors

Recent studies have increasingly highlighted the role of β3-ARs in retinal vascularization.

In the OIR model, β3-ARs are markedly upregulated in the retina during the hypoxic proliferative phase (28, 80), and their involvement in vascularization was first suggested by their coupling with nitric oxide (NO) production and VEGF induction, initially observed in mouse retinal explants (81). Early on, it was hypothesized that both β2- and β3-ARs might contribute to pathological angiogenesis, and that antagonism of either receptor subtype could exert antiangiogenic effects in OIR (82). However, while β2-AR antagonism effectively suppressed pathological neovascularization, β3-AR blockade showed no comparable efficacy, suggesting that β3-ARs are not critically involved in pathological angiogenesis (29).

Conversely, accumulating evidence points to a role for β3-ARs in physiological vascularization. During intrauterine life, β3-ARs represent the most abundantly expressed adrenergic receptor subtype in the retina, and their expression decreases markedly after birth, most likely as a consequence of increased oxygen exposure (14). This observation is consistent with the well-established notion that β3-ARs are tightly regulated by hypoxia (83–85), and more recently with the demonstration that their gene expression is directly controlled by HIF-1 (86). The decline in β3-AR expression following exposure to a more oxygen-rich environment strongly supports the view that β3-ARs are modulated by oxygen availability (87).

This expression pattern, opposite to that of β2-ARs, suggests that β3-ARs may sustain physiological vascularization, whereas β2-ARs may drive pathological angiogenesis. Further support for this hypothesis comes from studies in genetically modified mice lacking β1- and β2-ARs but retaining β3-AR expression. β1- and β2-AR knockout mice were markedly protected in the OIR model, showing substantially attenuated vascular regression during hyperoxia and a physiological pattern of revascularization during hypoxia, without evidence of pathological neovascularization. These findings support the notion that robust β3-AR expression preserves normal vascular growth against oxygen-induced injury (88).

**FIGURE 1:**
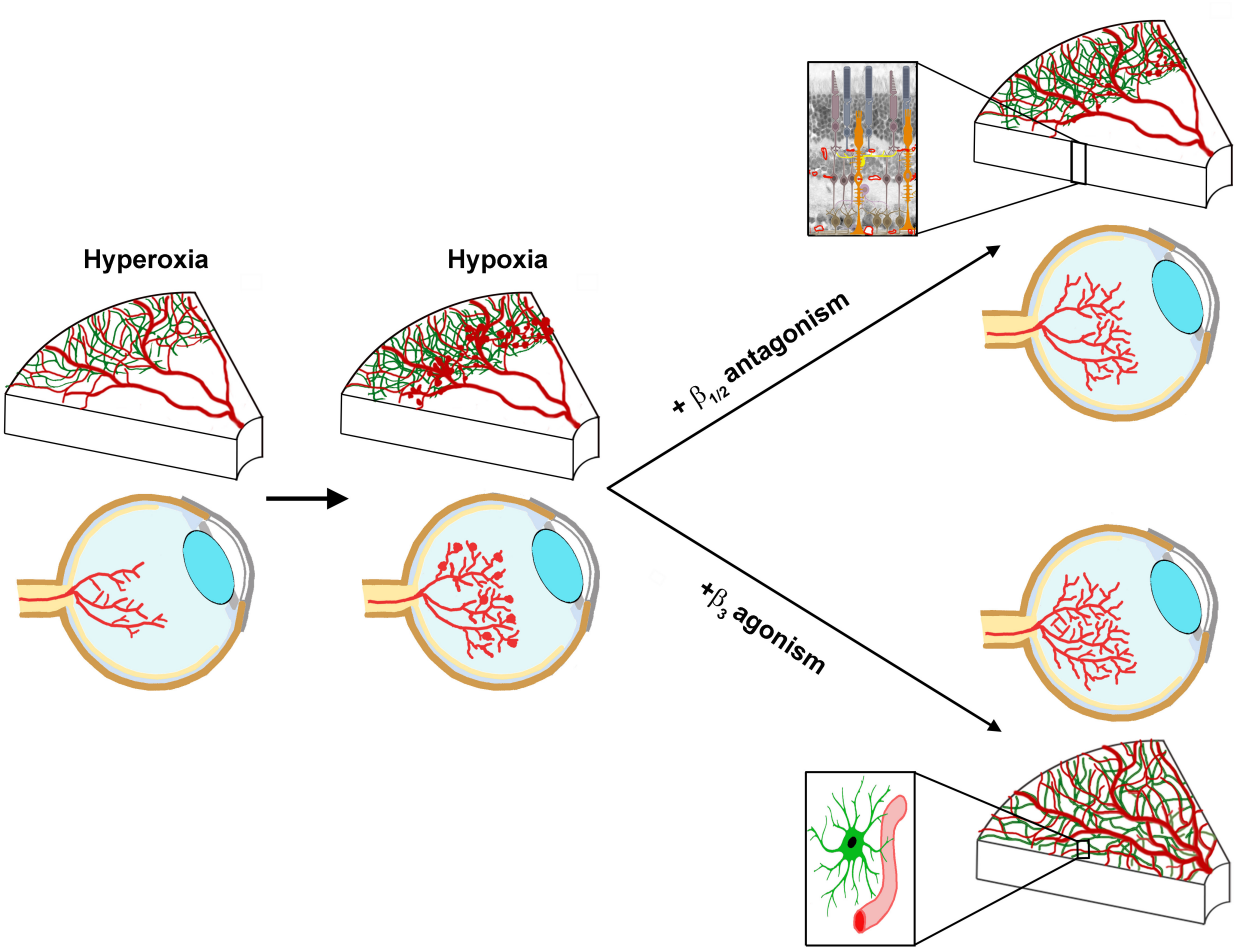
Resolved abnormal retinal vessel growth by β2-AR antagonism or β3-AR agonism. In the OIR model, hyperoxia-induced vaso-obliteration in the central retina leads to hypoxic environment that results in abnormal vessel proliferation in the mid-periphery. Reduced oxygen tension leads to sympathetic over-drive, which results in the release of norepinephrine (NE) that activates both β2-ARs expressed by Müller cells (enlarged magnification of the boxed area through the retinal thickness) and β3-ARs expressed by proliferating vessels. Both β2-AR antagonism and β3-AR agonism have been shown to counteract retinal vessel proliferation with different mechanisms. Antagonism of β2-ARs would prevent NE coupling to its receptors thus inhibiting the angiogenic drive in the midperipheral retina despite persisting vaso-obliteration in the central area. Activation of β3-ARs, instead, leads to the revascularization of the central retina otherwise rendered avascular by the first hyperoxic phase. This would occur through reconstitution of the astrocyte-guided formation of the vascular network (astrocyte/vessel recruitment in the enlarged magnification).

Our preliminary results provide new evidence that reinforces the idea that β3-ARs are actively involved in promoting physiological vascularization. Administration of a selective β3-AR agonist to mice subjected to OIR during the proliferative phase was preliminary found to restore physiological vascularization of the central retina - typically suppressed by hyperoxia - effectively counteracting the damage induced by excessive oxygen exposure. Restoration of physiological vascularization in the central retina, in turn, would prevent the subsequent development of pathological neovascularization. Notably, recovery of physiological vascularization would be associated with reconstitution of the astrocytic population in the central retina, thereby supporting that β3-AR activation may orchestrate an orderly, scaffold-guided vascularization that closely recapitulates normal vascular pattern (Figure 1).

The possibility that β3-AR activation might induce astrocyte recruitment and assembly into the retinal astrocytic template is further supported by the fact the norepinephrine (NE), which is released by the sympathetic nerve terminals in response to hypoxic conditions (28, 29), exerts a well-established differentiative role. NE, in fact, is known to promote the maturation of astrocytes through sympathetic overstimulation and β-adrenergic receptor signaling (89), including β3-ARs, which are highly expressed in primary astrocyte cultures (90). Based on these observations, several putative mechanisms can be hypothesized and will likely represent the focus of future investigations. First, it will be important to determine whether β3-AR agonism modulates the expression of PDGF-A in retinal ganglion cells or the expression of PDGFRα in astrocytes, both of which are critical regulators of astrocyte-mediated vascular guidance. Similarly, a potential relationship between pharmacological modulation of β3-AR activity and the expression of key astrocytic transcription factors, such as PAX2 and SOX9, warrants further exploration. An additional possibility is that activation of β3-ARs expressed by astrocytes may promote a metabolic rescue that enhances their survival. In this regard, evidence showing that NE increases intracellular glutathione levels in astrocytes through β3-AR stimulation suggests that pharmacological activation of β3-ARs could increase astrocytic resistance to oxidative stress (91). Another mechanism deserving consideration relates to the well-established role of β3-ARs in regulating lipid metabolism through the stimulation of lipolysis (92). This activity could promote the availability of fatty acids to retinal astrocytes, which are essential for maintaining astrocyte survival and function (93). Moreover, β3-AR activation has been shown to enhance cellular glucose uptake in astrocytes, a process that may further support astrocytic viability (94). Increased glucose uptake may occur within a broader β3-AR-driven metabolic shift toward glycolysis, accompanied by the induction of mitochondrial dormancy. This metabolic reprogramming, which renders cells less vulnerable to ischemic and hypoxic stress, resembles the Warburg-like phenotype observed in embryonic tissues and cancer cells (95). Finally, an additional hypothesis to be explored concerns the ability of β3-AR activation to promote the recruitment of cells with high stemness potential. Since this phenomenon has been described in tumor biology (85), it is conceivable that pharmacological stimulation of β3-ARs may also contribute to the recruitment of astrocytic precursors, thereby supporting astrocyte repopulation and physiological vascular patterning.

In line with the possibility that β3-AR activation promotes the revascularization of the central retina, astrocyte-derived key factors guiding endothelial cell organization need a detailed analysis. Indeed, available evidence indicates that β3-AR agonism induces the upregulation of VEGF (81), the central mediator of angiogenic signaling. In this context, VEGF production has been shown to originate, at least in part, from Müller glial cells as well as from retinal endothelial cells themselves (96). However, during the early differentiative phase of retinal development, when the tissue is still largely avascular, astrocytes represent the predominant source of VEGF (13). Accordingly, it is plausible that β3-AR agonism would act in a coordinated manner, not only by promoting astrocyte recruitment and assembly into the astrocytic template, but also by activating the secretion of pro-angiogenic factors that subsequently would guide endothelial cell migration and organization.

In summary, the adrenergic system plays a central role in regulating retinal angiogenesis under hypoxic conditions. During intrauterine life, β3-ARs predominate, and their activation - together with pro-angiogenic signaling and astrocytic scaffold recruitment – seems to drive physiological vascularization. After birth, β2-ARs replace β3-ARs as the dominant receptors. In the presence of hypoxia, this shift preserves the angiogenic drive but, in the absence of astrocytic involvement, results in disorganized pathological vascularization.

## Therapeutic perspectives and the concept of a pharmacological artificial placenta

**FIGURE 2:**
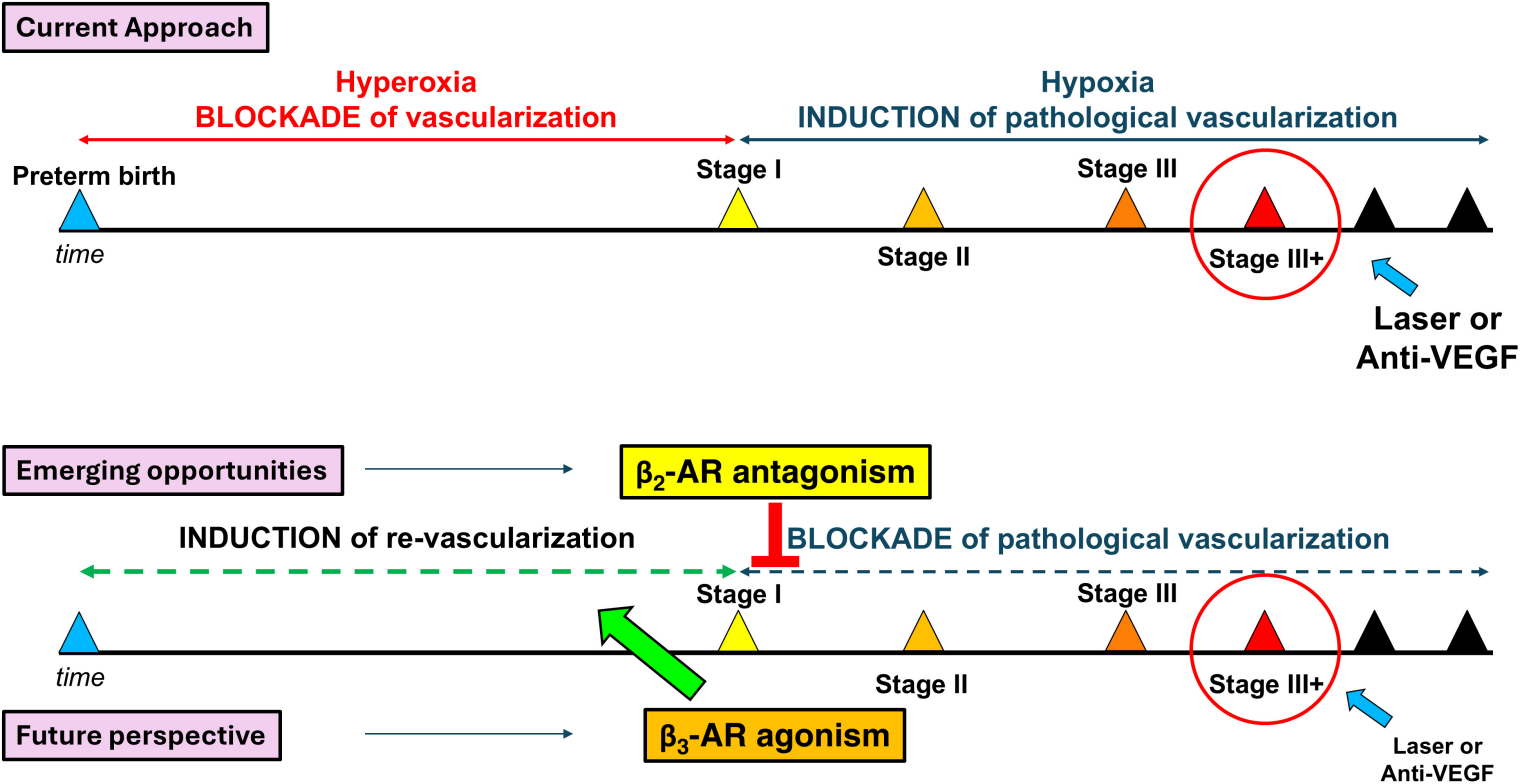
Schematic representation of current and emerging therapeutic strategies for ROP. The current approach focuses on preventing retinal detachment (top). Available pharmacological options (bottom) counteract pathological neovascularization through non-selective β1- and β2-AR antagonists. A future perspective is the use of β3-AR agonists in humans, aiming to prevent pathological neovascularization by restoring a more physiological vascularization.

These observations lay the foundation for novel therapeutic opportunities in the management of ROP and place β-adrenergic receptor–targeted approaches within a broader therapeutic framework that also includes emerging non-adrenergic strategies, such as omega-3 polyunsaturated fatty acid supplementation, which primarily modulates inflammatory and oxidative pathways to indirectly limit pathological neovascularization (97). In contrast, β-AR-based interventions act more directly on neurovascular and glial-vascular signaling. At present, ophthalmic propranolol is available - albeit only as a galenic formulation - to counteract pathological neovascularization, and it is conceivable that, in the near future, other commercially available non-selective β-blockers may also enter clinical application. However, the most compelling and innovative future perspective in ROP therapy lies in promoting physiological retinal vascularization through pharmacological stimulation of β3-ARs. This strategy holds the potential not only to restore areas of hyperoxia-induced vascular regression with physiological vasculature, but also to prevent the compensatory development of pathological neovascularization, thereby addressing a long-standing unmet need in ROP treatment (Figure 2).

This approach is embedded within a broader and more ambitious framework aimed at recreating intrauterine-like conditions through pharmacological mimicry of the fetal environment—a paradigm we have termed the “*pharmacological artificial placenta*” (22).

Based on the observation that β3-ARs exert their primary physiological functions during intrauterine life and are downregulated upon oxygen exposure at birth, we hypothesized that many major complications of prematurity could be at least partly attributed to the loss of β3-AR-mediated protective signaling. Premature birth, occurring when retinal vascularization is still incomplete, may compromise physiological angiogenesis through multiple mechanisms, with β3-AR downregulation representing one potential contributor. This raises the possibility that β3-AR-dependent protective mechanisms could be pharmacologically reactivated through agonist stimulation. From this rationale, we envisioned that early treatment with β3-AR agonists could counteract the deleterious effects of hyperoxia.

Experimental data are encouraging. In neonatal rats exposed to high oxygen concentrations, early treatment (initiated from postnatal day 1) with β3-AR agonists significantly attenuated damage in the colon (98) and ileum (99), protecting not only vascular structures (99) but also the enteric nervous system (100); more recent evidence further supports the protective role of β3-AR activation under hyperoxic conditions, as pharmacological β3-AR agonism has been shown to protect the lung from hyperoxia-induced injury while simultaneously promoting a partial recovery of physiological vascularization (101). By contrast, in neonatal mice subjected to the OIR model, the benefits of β3-AR agonists were observed only during the proliferative phase, not during the initial hyperoxic phase. This discrepancy may reflect differential receptor expression dynamics: in mice, hyperoxia appears to abolish β3-AR expression in the retina, rendering early treatment ineffective, whereas in rats, β3-AR expression in the intestine is downregulated but not fully suppressed by hyperoxia, allowing agonist stimulation to remain effective. Thus, heterogeneity in treatment response may depend on tissue-specific susceptibility or interspecies variability in β3-AR regulation.

Despite these differences, the overall evidence from preclinical studies across multiple models consistently indicates that β3-AR agonist treatment can prevent or mitigate the damage typically induced by hyperoxic exposure. In the retina, pharmacological activation of β3-ARs preserves physiological angiogenesis and sustains astrocyte survival, ultimately ensuring orderly vascular development even under adverse postnatal oxygen conditions. This strategy represents an exciting and challenging frontier in ROP therapy, aimed not merely at preventing retinal detachment but at actively guiding vascular development along its physiological trajectory. By leveraging the protective potential of β3-ARs, it may be possible to achieve a paradigm shift in the treatment of ROP, restoring developmental mechanisms that are otherwise disrupted by premature birth.

Although clinical data on the use of β3-AR agonists in neonates are still lacking, the ultimate aim of this research field is to determine, in human preterm infants, whether the administration of such agents might facilitate the restoration of physiological retinal vascularization and thereby reduce the risk of pathological angiogenesis (22, 102). If future studies confirm that β3-AR activation selectively promotes physiological angiogenesis, while β2-AR activation drives pathological neovascularization, an even more forward-looking therapeutic approach could involve a dual strategy: coupling β3-AR agonism, to enhance para-physiological mechanisms, with non-selective β2-AR antagonism, to prevent the development of aberrant vascular tufts. While these perspectives may appear somewhat speculative at present, it is worth underlining that β3-AR agonists are already widely employed in humans - for indications unrelated to retinopathy - with excellent safety profiles and minimal adverse effects. The most promising translational pathway would therefore mirror the trajectory followed for propranolol, moving step by step from experimental animal studies to clinical application.

As to the therapeutic perspective of propranolol, several important questions remain open and warrant further investigation. For instance, the optimal dosage for topical propranolol administration has not been definitively established yet. It cannot be excluded that concentrations higher than the currently used 0.2% formulation may provide greater efficacy while maintaining an acceptable safety profile (16). A similar dose-finding pathway will likely be required for other non-selective β-blockers, should preclinical studies yield favorable results. With regard to β3-AR agonist-based approaches, the unresolved preclinical issues limit for now the definition of timing a dosage strategy. At present, β3-AR agonists are approved for human use exclusively via oral administration, and their potential application in preterm neonates will require careful evaluation of the most appropriate route of delivery, as well as the identification of safe and effective dosing regimens. A particularly critical aspect concerns the optimal timing of β3-AR agonist administration. While non-selective β-blockers, which counteract pathological neovascularization, have a clear rationale for use during the proliferative phase of ROP, when pathological angiogenesis is already initiated (16), the timing for β3-AR agonist treatment remains less well defined. On the one hand, an earlier administration during the ischemic phase of ROP might be envisioned to prevent the regression of physiological vascularization. On the other hand, the effectiveness of β3-AR agonists is likely to depend on receptor availability, suggesting that these agents may exert their maximal effects when β3-AR expression increases as under hypoxia. In this context, preclinical studies in mice showing a marked upregulation of β3-ARs during the proliferative phase support the hypothesis that β3-AR agonist treatment may be more effective when administered during the hypoxic phase (28, 80).

However, the expected benefits of a treatment with β3-ARs agonists would extend beyond the prevention/treatment of ROP alone, potentially encompassing the mitigation of other major prematurity-related morbidities, including BPD, necrotizing enterocolitis, and brain injury (22, 102).

## Conclusion

In conclusion, progress in the treatment and prevention of ROP has thus far been limited and, in many respects, disappointing. One possible explanation for these modest achievements lies in the prevailing research approach: diseases are often investigated primarily through their clinical manifestations, and therapeutic strategies are then designed to “patch” the observed defects. In the case of ROP, this has meant that most neonatologists and ophthalmologists begin to focus on the disease only once it has already become clinically relevant, with little attention - and virtually no capacity for intervention - during the preclinical phase.

Yet the disorders typically associated with prematurity differ fundamentally from other diseases. Preterm infants - although at high risk of developing severe morbidities - are not intrinsically ill. Rather, they are infants who, had they remained *in utero*, would not have developed any of the conditions associated with prematurity. What we call “diseases” in this context are, in fact, maladaptive responses to an extrauterine environment encountered too early, responses that might otherwise represent normal biological adaptations at a later stage of development. For this reason, it becomes essential not only to investigate why preterm infants may develop such severe complications after birth, but also to ask why they thrive so well while still in the womb. In other words, we must shift our attention toward a deeper understanding of the physiology of fetal well-being and of strategies that could help to reactivate this state after preterm delivery.

The road ahead is undoubtedly long, and the biological mechanisms underlying physiological intrauterine development are numerous and complex. Nevertheless, this is the direction in which meaningful progress must be sought. Identifying a role for β3-ARs represents only a very small step, but one that may point toward a more incisive approach to conditions we continue to label as prematurity-related “diseases.”
